# Loss of MBNL1-mediated retrograde BDNF signaling in the myotonic dystrophy brain

**DOI:** 10.1186/s40478-023-01540-x

**Published:** 2023-03-15

**Authors:** Pei-Ying Wang, Ting-Yu Kuo, Lee-Hsin Wang, Wen-Hsing Liang, Guey-Shin Wang

**Affiliations:** 1 Institute of Biomedical Sciences, Academia Sinica, 128, Section 2, Academia Rd. Nangang, Taipei, 115 Taiwan; 2 Taiwan International Graduate Program in Molecular Medicine, National Yang Ming Chiao Tung University and Academia Sinica, Taipei, Taiwan; 3 Taiwan International Graduate Program in Interdisciplinary Neuroscience, National Yang Ming Chiao Tung University and Academia Sinica, Taipei, Taiwan

**Keywords:** Myotonic dystrophy, MBNL1, BDNF, DYNLL2, Retrograde transport

## Abstract

Reduced brain volume including atrophy in grey and white matter is commonly seen in myotonic dystrophy type 1 (DM1). DM1 is caused by an expansion of CTG trinucleotide repeats in the 3’ untranslated region (UTR) of the Dystrophia Myotonica Protein Kinase (*DMPK*) gene. Mutant *DMPK* mRNA containing expanded CUG RNA (*DMPK*-CUG^exp^) sequesters cytoplasmic MBNL1, resulting in morphological impairment. How *DMPK*-CUG^exp^ and loss of MBNL1 cause histopathological phenotypes in the DM1 brain remains elusive. Here, we show that BDNF-TrkB retrograde transport is impaired in neurons expressing *DMPK*-CUG^exp^ due to loss of cytoplasmic MBNL1 function. We reveal that mature BDNF protein levels are reduced in the brain of the DM1 mouse model EpA960/CaMKII-Cre. Exogenous BDNF treatment did not rescue impaired neurite outgrowth in neurons expressing *DMPK*-CUG^exp^, whereas overexpression of the cytoplasmic MBNL1 isoform in *DMPK*-CUG^exp^-expressing neurons improved their responsiveness to exogenous BDNF. We identify dynein light chain LC8-type 2, DYNLL2, as an MBNL1-interacting protein and demonstrate that their interaction is RNA-independent. Using time-lapse imaging, we show that overexpressed MBNL1 and DYNLL2 move along axonal processes together and that MBNL1-knockdown impairs the motility of mCherry-tagged DYNLL2, resulting in a reduced percentage of retrograde DYNLL2 movement. Examination of the distribution of DYNLL2 and activated phospho-TrkB (pTrkB) receptor in EpA960/CaMKII-Cre brains revealed an increase in the postsynaptic membrane fraction (LP1), indicating impaired retrograde transport. Finally, our neuropathological analysis of postmortem DM1 tissue reveals that reduced cytoplasmic MBNL1 expression is associated with an increase in DYNLL2 and activated pTrkB receptor levels in the synaptosomal fraction. Together, our results support that impaired MBNL1-mediated retrograde BDNF-TrkB signaling may contribute to the histopathological phenotypes of DM1.

**Supplementary Information:**

The online version contains supplementary material available at 10.1186/s40478-023-01540-x.

## Introduction

Myotonic dystrophy type 1 (DM1), also known as Steinert’s disease, is the most common form of adult-onset muscular dystrophy [1]. Cognitive deficits are commonly seen in individuals with DM1, including mental retardation, anxiety, depression, autism spectrum disorder, psychiatric disorders and neurodegeneration [2]. These behavioral, psychological and cognitive impairments are correlated with structural and connectivity changes in various brain regions [3, 4]. DM1 is caused by an expansion of CTG repeats in the 3’ untranslated region (UTR) of the Dystrophia Myotonica Protein Kinase (*DMPK*) gene. Nuclear *DMPK* mRNAs containing expanded CUG repeats bind and sequester MBNL proteins, resulting in loss of the cytoplasmic and nuclear functions of MBNL proteins [5, 6]. Both MBNL1 and 2 are required for neuronal maturation; cytoplasmic-localized MBNL1 promotes neurite outgrowth [7], whereas nuclear-localized MBNL2 regulates the developmental transition associated with alternative splicing and polyadenylation [8]. Loss of MBNL1 and 2 functions elicits abnormal neurotransmission and cognitive behaviors, indicating roles in regulating synaptic transmission [9, 10].

A brain-specific DM1 mouse model EpA960/CaMKII-Cre that expresses expanded CUG RNA in neurons of the postnatal brain recapitulates features of the DM1 brain, including learning disability, misregulation of alternative splicing, and neurodegeneration [11]. Though sequestration of MBNL proteins results in loss of MBNL functions, expression of expanded CUG RNA in neurons was actually found to induce sequential alterations of MBNL proteins, with reduced expression of cytoplasmic MBNL1 due to deubiquitination-mediated nuclear translocation being followed by neurodegeneration-induced calpain-mediated MBNL2 degradation [7, 8]. Despite it being known that reduced cytoplasmic MBNL1 expression occurs prior to axon and dendrite degeneration in the EpA960/CaMKII-Cre brain and that overexpression of cytoplasmic MBNL1 in neurons expressing expanded CUG RNA can ameliorate the morphological defects, it remains unclear how reduced cytoplasmic MBNL1 leads to morphological impairment.

Cognitive impairment accompanied by structural changes arising from defects in neuronal morphology and connectivity is a common feature of neurological diseases [12, 13]. The causal mechanisms of histopathological abnormalities associated with aberrant connectivity remain elusive, yet altered expression of brain-derived neurotrophic factor (BDNF) and perturbed BDNF signaling are commonly reported for several neurological diseases, including Alzheimer’s disease (AD), Parkinson’s disease (PD), Huntington’s disease (HD), and amyotrophic lateral sclerosis (ALS) [14, 15]. BDNF plays important roles in promoting neurite development, neuronal survival and long-term maintenance of synapse integrity and dendrite maintenance [16], rendering BDNF an appropriate therapeutic target for neurological disease [17]. A reduction of BDNF level in the serum of DM1 patients has been reported previously [18], but the level of BDNF and the integrity of BDNF signaling in DM1 brains have not been established nor is it known if BDNF supplementation may improve the synaptic activity of DM1 neurons.

In the present study, we determine if BDNF signaling remains intact in the DM1 brain and explore how reduced cytoplasmic MBNL1 affects neuronal morphology. We show that the level of mature BDNF protein is reduced in EpA960/CaMKII-Cre mouse brain. We test the impact of exogenous BDNF in neurons expressing expanded CUG RNA or those displaying MBNL1 knockdown, revealing loss of BDNF responsiveness in expanded CUG RNA-expressing neurons or MBNL1-knockdown neurons. We also show that MBNL1 interacts with dynein light chain LC8-type 2, DYNLL2, and this interaction is required for the retrograde BDNF-TrkB transport mediated by DYNLL2. MBNL1 knockdown in cultured hippocampal neurons limited retrograde movement of DYNLL2, which is consistent with increased DYNLL2 and activated phospho-TrkB (pTrkB) receptor levels in the postsynaptic membrane fraction (LP1) of EpA960/CaMKII-Cre brains. Similarly, we observed that reduced MBNL1 cytoplasmic expression and increased DYNLL2 and pTrkB receptor expressions in the synaptosomal fraction, together supporting impaired retrograde BDNF-TrkB transport. Thus, we provide insights into how loss of cytoplasmic MBNL1 function may contribute to the histopathological phenotypes of DM1.

## Materials and methods

### Animals

Establishment of the EpA960/CaMKII-Cre line has been described previously [11]. Animals in the C57BL6 background were maintained on a standard 12-h light/dark cycle with the light on at 8:00 AM. Food and water were available *ad libitum*. Brain samples used for protein extraction were obtained from littermates of both genders. All animal experiments were performed with the approval of the Academia Sinica Institutional Animal Care and Utilization Committee (IACUC, Academia Sinica, Taiwan).

### Human postmortem brain samples

All postmortem brain tissues, i.e., three DM1 and three age-matched non-DM1 human brains (four males and two females), were obtained from the NICHD Brain and Tissue Bank for Developmental Disorders (University of Maryland, MD, USA). Frozen tissues were subjected to biochemical fractionation, or underwent cryo-sectioning at 10-µm thickness for immunofluorescence staining and in situ hybridization. The protocol was approved by the Institutional Review Board, Academia Sinica, Taiwan.

### Primary hippocampal neuron culture and transfection

Rat hippocampal neurons were dissociated from embryos at embryonic day 18–19 as described [7]. Briefly, hippocampal tissues were trypsinizied with 0.5X trypsin in Hank’s buffered salt solution, resuspended in growth medium (neurobasal medium supplemented with 2% B27 supplement, 0.5 mM glutamine, and 12.5 mM glutamate), and plated in 12-well plates containing glass coverslips coated with poly-L-lysine (1 mg/ml) at 200,000 neurons per well to examine the morphological features of axons. Transfection was performed at day 2 or 3 of in vitro culture (DIV) to assess axonal outgrowth by calcium phosphate precipitation. Two to three days after transfection (i.e., 5 DIV), neurons were harvested for morphological assessment based on their co-expression of GFP plasmid.

### Immunofluorescence, immunohistochemistry, fluorescence in situ hybridization and quantification

For immunofluorescence staining, cells were fixed with 4% PFA and 4% sucrose in PBS for 15 min at room temperature 2–3 days after transfection, followed by permeabilization with 0.2% Triton X-100 in PBS for 15 min. After blocking with 3% normal goat serum (NGS), cells were incubated with primary antibodies diluted in PBS containing 1% NGS at 4 °C overnight, followed by PBS washes. The cells were then incubated with secondary antibodies conjugated with Alexa Fluor 488 and/or 594 (Invitrogen) for 2 h.

To detect RNA foci on postmortem human brain sections, fluorescence in situ hybridization was performed as described [7, 11]. Briefly, sections were post-fixed with 4% PFA in PBS, followed by UV cross-linking, permeabilization, DNase treatment, prehybridization, and hybridization with Cy3-labeled (CAG)_7_ LNA probe at 42 °C for 2 h. For the colocalization of MBNL1 foci with RNA foci, after in situ hybridization, sections were incubated in blocking solution (3% NGS in 1X PBS containing 0.02% Triton X-100) for 1 h at room temperature, and incubated with anti-MBNL1 and anti-MAP2 antibodies. After washing, sections were incubated with secondary antibody conjugated with Alexa Fluor 488 and 647 (Invitrogen). Fluorescent images were acquired under a fluorescent microscope (AxioImager M2, Carl Zeiss) equipped with a 20x/0.8 or 40x/0.75 objective lens or a confocal laser scanning microscope (LSM700, Carl Zeiss) equipped with a 40x/1.4/oil objective lens or a 63x/1.4/oil objective lens.

For immunohistochemistry, vibratome sections collected from mouse brains were used as described with modifications [11]. Briefly, brain sections were treated with 3% H_2_O_2_ to remove endogenous peroxidase activity, followed by permeabilization, blocking and incubation with primary antibody. After wash, sections were incubated with biotinylated secondary antibody (Vector Laboratories) for 2 h, then with ABC reagent (Vector Laboratories) for 2 h, followed by signal development using the SG substrate kit (Vector Laboratories). Sections were dehydrated and cleared by xylene, and cover-slipped with permount medium (Vector Laboratories). Images were acquired under a upright microscope (BX51, Olympus) equipped with a 10x/0.4 or 20x/0.75 objective lenses.

To quantify neuronal morphology, the lengths of all axonal branches were determined in ImageJ v.1.45 (NIH) to represent total axon length. All plots show data from three independent transfections, with 15–30 neurons assessed for each transfection. To examine the distribution of TrkB receptor in mouse brain, we first defined proximal neurite region that includes soma region and neurite extended from soma in 100 µm length. We then measured the intensity of TrkB immunoreactivity in the proximal neurite region of neurons in cortical layer V. For each animal, 5–6 image frames and 20 neurons were collected. To quantify the TrkB immunoreactivity in postmortem human brain sections, only the intensity in the cell body region was measured.

### Plasmids

The *Mbnl1*-specific shRNA clone (pLKO_219085, TGACAGCACAATGATTGATAC) and *Dynll2*-specific shRNA clone (pLKO_71774, GAAGGACATTGCTGCCTATA) targeting mouse MBNL1 and DYNLL2, respectively, were purchased from the RNAi Core Facility of Academia Sinica (Taiwan). Plasmids expressing *Dmpk* exons 11–15 with no CUG repeats (*Dmpk*-CUG^0^) or with 960 interrupted CTG repeats (*Dmpk*-CTG^960^) [19] were kindly provided by Dr. Thomas Cooper (Baylor College of Medicine, Houston, TX). The fluorescence tag-conjugated DYNLL2 and MBNL1 plasmids were subcloned in-house.

### BDNF treatment

To determine the responses of neurons expressing *Dmpk*-CUG^960^ mRNA to BDNF treatment, they were transfected at 3 DIV and then treated 1 day later with BDNF (100 ng/ml, Peprotech) for 24 h before undergoing morphological analysis [20]. To determine the effect of MBNL1 or DYNLL2 knockdown on BDNF responses, cultured hippocampal neurons were transfected at 2 DIV and incubated for a further 2 days (4 DIV), before being treated with BDNF for another 24 h prior to characterization. For live-cell recording, treated with BDNF prior to the recording for 30 min at 5 DIV.

### Antibodies

Antibodies used included anti-GFP (A11122, 1:1000, Invitrogen) to label transfected cells and neuronal morphologic features. The antibodies used for Western blot analyses include: anti-BDNF (ab108319, 1:3000, Abcam), anti-FLAG M2-peroxidase conjugated (1:20,000, Sigma), anti-GFP-peroxidase conjugated (clone 3D8A1B8, 1:10,000, Abking), anti-MBNL1 polyclonal antibody (ABE241, 1:1000, Millipore) [21], anti-DIC (dynein intermediate chain)(ab23905, 1:1000, Abcam), anti-DYNLL2 (Merck, ABT140), anti-PSD95 (post-synaptic density 95, clone 3H4.3, 1:2000, Millipore), anti-synaptophysin (S5768, 1:1000, Sigma), anti-TrkB (#4603, 1:1000, Cell Signaling), anti-phospho-TrkB (Y817) (MA5-32,207, 1:1000, Invitrogen), anti-HSP90 (heat shock protein 90, a gift from Dr. Yi-Ping Hsueh, 1:2000) and anti-GAPDH (MAB374, 1:20,000, Millipore). Anti-GFP antibodies (clones 19C8 and 19F7) used in immunoprecipitation were from Memorial Sloan-Kettering Monoclonal Antibody Facility. To detect the level of BDNF, the nitrocellulose membrane underwent antigen retrieval to boost the signal [22].

### Subcellular fractionation of mouse and postmortem human brains

The cortices and hippocampi from adult mouse brains (3 months old, C57B6/J) or the brains of different mouse genotypes (control and EpA960/CaMKII-Cre, 1 to 1.5 years old) were collected for subcellular fractionation as described previously [23]. The frozen tissues were dounced in lysis buffer (320 mM sucrose, 4 mM HEPES, 2 mM DTT, 2 mM MgCl_2_, 1 mM EDTA, and proteinase inhibitor) and collected as homogenate (H), before undergoing centrifugation at 800 g to remove nuclei and other large debris (P1). The supernatant was centrifuged at 9,200 g to obtain a crude synaptosomal fraction (P2), which was then lysed with hypotonic buffer and centrifuged at 25,000 g to pellet a synaptosomal membrane fraction (LP1). The supernatant (LS1) was then centrifuged at 165,000 g to obtain a crude synaptic vesicle fraction (LP2) and a soluble fraction (LS2). The supernatant above the P2 fraction (S2) was then centrifuged at 165,000 g to obtain a cytosolic soluble fraction (S3) and a light membrane fraction (P3). A total of 5 µg of protein extract from the LP1, LP2, and LS2 fractions and 20 µg of protein extract from each fraction were subjected to Western blot analysis. The frozen human brain tissues were sliced into 0.5 cm^3^ pieces and then underwent the same procedure as described above for mouse brains.

### Live-cell imaging

For time-lapse imaging of hippocampal neurons, cells were plated on 30 mm-diameter glass-bottom dishes (WillCo-dish) at 200,000 neurons per well. To examine the mobility of MBNL1^ΔEx5^ or the effect of MBNL1 knockdown on DYNLL2 mobility, transfection was performed at 4 DIV, followed by time-lapse recording 1 day later. Time-lapse imaging was performed using a confocal microscope (Zeiss) equipped with a 37 °C incubator and 5% CO_2_ supply. Images were taken every 2.5 s for 20 min and analyzed in MetaMorph software. Kymograph analysis was conducted on a 100 μm-extent of primary axon located at least 80 μm from the soma. The movement events and movement types of MBNL1-mCherry or DYNLL2-mCherry in MBNL1-depleted neurons were determined by kymograph [24].

### Immunoprecipitation

To evaluate the interaction between MBNL1 and DYNLL2 or TrkB, cell lysates were prepared as described previously [25] with a minor modification. In brief, Neuro2A cells expressing FLAG-MBNL1 and GFP-DYNLL2 or EGFP-TrkB for 48 h were harvested in lysis buffer (25 mM Tris–HCl pH8.0, 50 mM NaCl, 0.5% Triton X-100, 5 mM DTT with protease inhibitors). The protein lysate was separated into two vials and 1 µl RNase was added into one of the vials and incubated at 37 °C for 1 h. Taking 1/10 of the protein lysate as input, the remainder was incubated overnight at 4 °C with agarose beads conjugated with 50 µg anti-GFP antibody or 1 µg anti-FLAG antibody. Proteins were eluted by adding SDS electrophoresis sample buffer (75 mM Tris–HCl pH 6.8, 3% SDS, 0.1% bromophenol blue, 15% glycerol, 40 mM DTT) and incubated at 55 °C before undergoing SDS-PAGE.

### Statistical analysis

Data are presented as means ± SEM and were analyzed in SigmaPlot 12.5 (Systat Software Inc.). To compare two groups, unpaired two-tailed Student’s *t*-tests were used. For comparison of more than two groups, one-way ANOVA tests were used, followed by Holm-Sidak multiple comparison tests. Test results with a *P* value of < 0.05 were considered statistically significant. No statistical methods were used to pre-determine the sample size. Data collection and analyses were not conducted blind. The experiments using mouse and patient tissues were not randomized. Only for each independent transfection in cultured neurons were cells on coverslips randomly allocated to subject for transfections.

## Results

### BDNF responsiveness is impaired in neurons expressing expanded CUG RNA or depleted of MBNL1

**Fig. 1:**
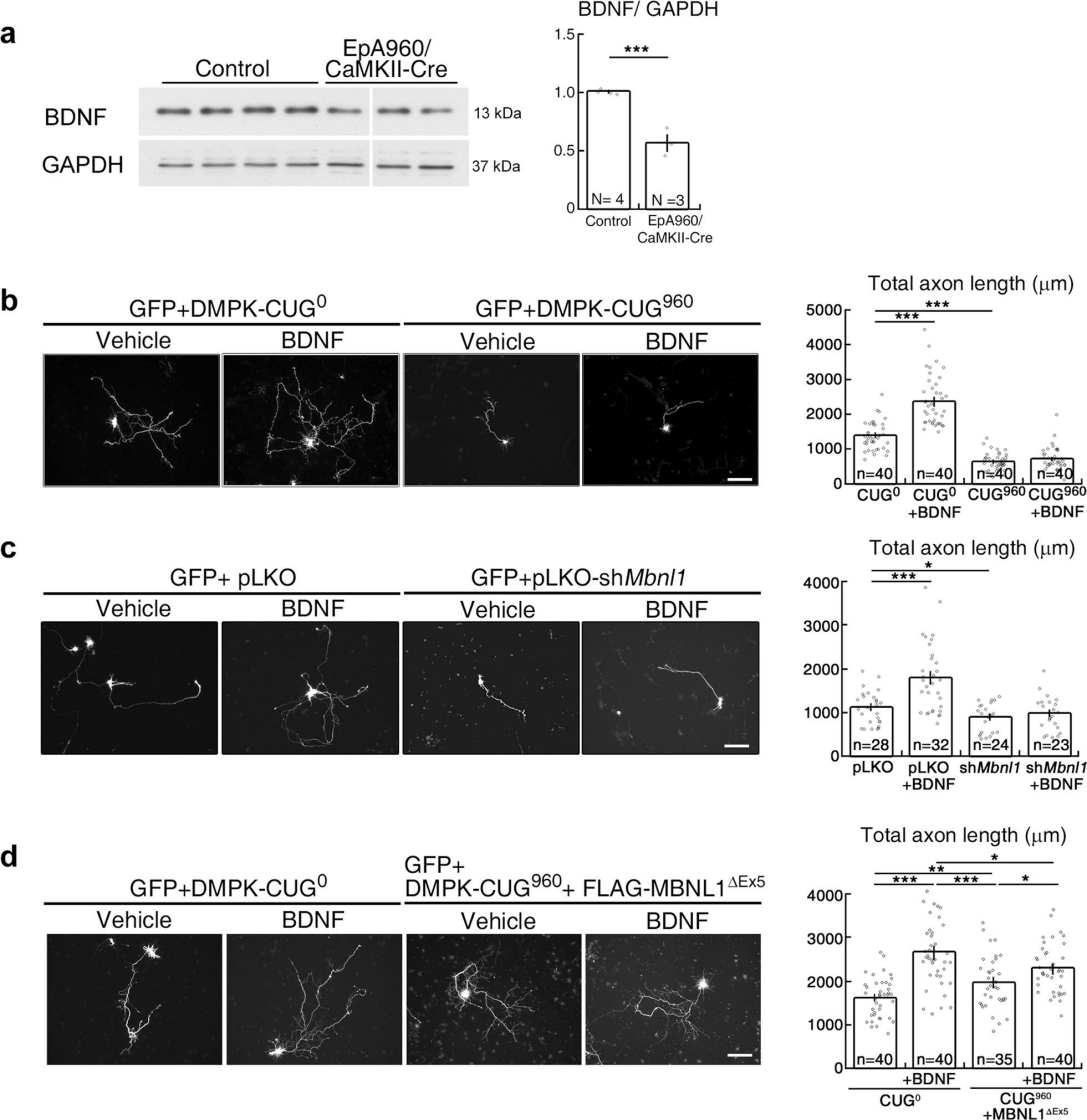
Loss of cytoplasmic MBNL1 expression impairs responsiveness to BDNF supplementation in neurons. (**a**) Examination of BDNF levels by Western blotting analysis in brains from control and EpA960/CaMKII-Cre mice aged one to 1.5 years. Three non-Tg and one EpA960 mice were included as controls. Quantification of BDNF levels, normalized with GAPDH, are shown at right. (**b, c**) Effect of BDNF treatment on axon development in neurons expressing *Dmpk*-CUG^960^ mRNA or upon MBNL1 depletion. (**d**) Effect of cytoplasmic MBNL1 overexpression on neuronal responsiveness to BDNF treatment. Neurons were transfected with plasmids expressing *Dmpk*-CUG^960^ mRNA (DMPK-CUG^960^) or *Dmpk* mRNA control (DMPK-CUG^0^) in (**b**), with plasmids expressing *Mbnl1* shRNA (pLKO-sh*Mbnl1*) or control shRNA (pLKO) in (**c**), or with plasmids expressing *Dmpk*-CUG^960^ plus cytoplasmic MBNL1 (FLAG-MBNL1^ΔEx5^) or *Dmpk* mRNA control alone in (**d**). Plasmid expressing GFP was co-transfected to label neuronal morphology. Quantification of total axon length is shown at right. Numbers of neurons (n, from three independent cultured neuronal preparations and transfections) used for quantification are indicated. Data are mean ± SEM. *p < 0.05, **p < 0.01, ***p < 0.001, by Student’s *t*-test (**a**) or one-way ANOVA with Holm-Sidak test (**b-d**). Scale bar: **b**-**d**, 100 μm

To determine if BDNF signaling is intact and BDNF supplementation could represent a possible therapeutic avenue for DM1, first we examined the levels of BDNF in EpA960/CaMKII-Cre mouse brains. We observed that the levels of mature BDNF protein were reduced in the brain of EpA960/CaMKII-Cre mice aged 1 to 1.5 years (Fig. 1a). We wondered if this reduction in BDNF may be the cause of morphological phenotypes in neurons expressing expanded CUG RNA and, consequently, if exogenous BDNF treatment might rescue the morphological impairment displayed by DM1 neurons. To test this possibility, we co-transfected a GFP-expressing construct with plasmid expressing a human *DMPK* 3’ UTR mRNA with or without 960 CUG repeats (DMPK-CUG^960^ and DMPK-CUG^0^, respectively) into cultured hippocampal neurons and then determined neuronal responsiveness to this BDNF treatment. We found that administration of BDNF to neurons expressing DMPK-CUG^0^ promoted axon outgrowth by increasing total axon length (Fig. 1b). However, neurons expressing DMPK-CUG^960^ did not respond to BDNF stimulation and still exhibited impaired axonal outgrowth. The morphological impairment of DMPK-CUG^960^-expressing neurons was likely due to loss of cytoplasmic MBNL1 function, so next we examined the responsiveness of MBNL1-depleted neurons to BDNF. Using a *Mbnl1*-specific short hairpin RNA (shRNA) construct to deplete MBNL1, we observed that MBNL1-knockdown neurons also failed to respond to BDNF treatment and exhibited impaired axonal outgrowth (Fig. 1c). Thus, BDNF signaling or BDNF responsiveness is impaired in neurons expressing CUG-repeat-expanded *Dmpk* mRNA or those depleted of MBNL1.

We previously showed that expressing excessive but not equal amounts of the cytoplasmic isoform of MBNL1 (MBNL1^ΔEx5^) could fully rescue the morphological phenotypes displayed by DMPK-CUG^960^-expressing neurons [7]. To test if MBNL1^ΔEx5^ plays a role in regulating BDNF responsiveness, we determined if MBNL1^ΔEx5^ could improve the BDNF responsiveness of DMPK-CUG^960^-expressing neurons. To do so, we co-transfected neurons with equal amounts of plasmids expressing DMPK-CUG^960^ and MBNL1^ΔEx5^, then subjected them to BDNF stimulation, and finally examined their morphologies. Expression of equal amounts of DMPK-CUG^960^ and MBNL1^ΔEx5^ only partially rescued the morphological phenotypes (Fig. 1d). Upon applying BDNF stimulation, expression of MBNL1^ΔEx5^ further enhanced the total axon length of neurons expressing DMPK-CUG^960^ relative to MBNL1-replenished neurons also expressing DMPK-CUG^960^ but that did not undergo BDNF stimulation (Fig. 1d). Extension of total axonal length was not as pronounced as for DMPK-CUG^0^-expressing neurons, likely because a fraction of MBNL1^ΔEx5^ was still sequestered into the nucleus. These results indicate that MBNL1^ΔEx5^ may regulate BDNF signaling.

### MBNL1 interacts with the cytoplasmic dynein DYNLL2

**Fig. 2:**
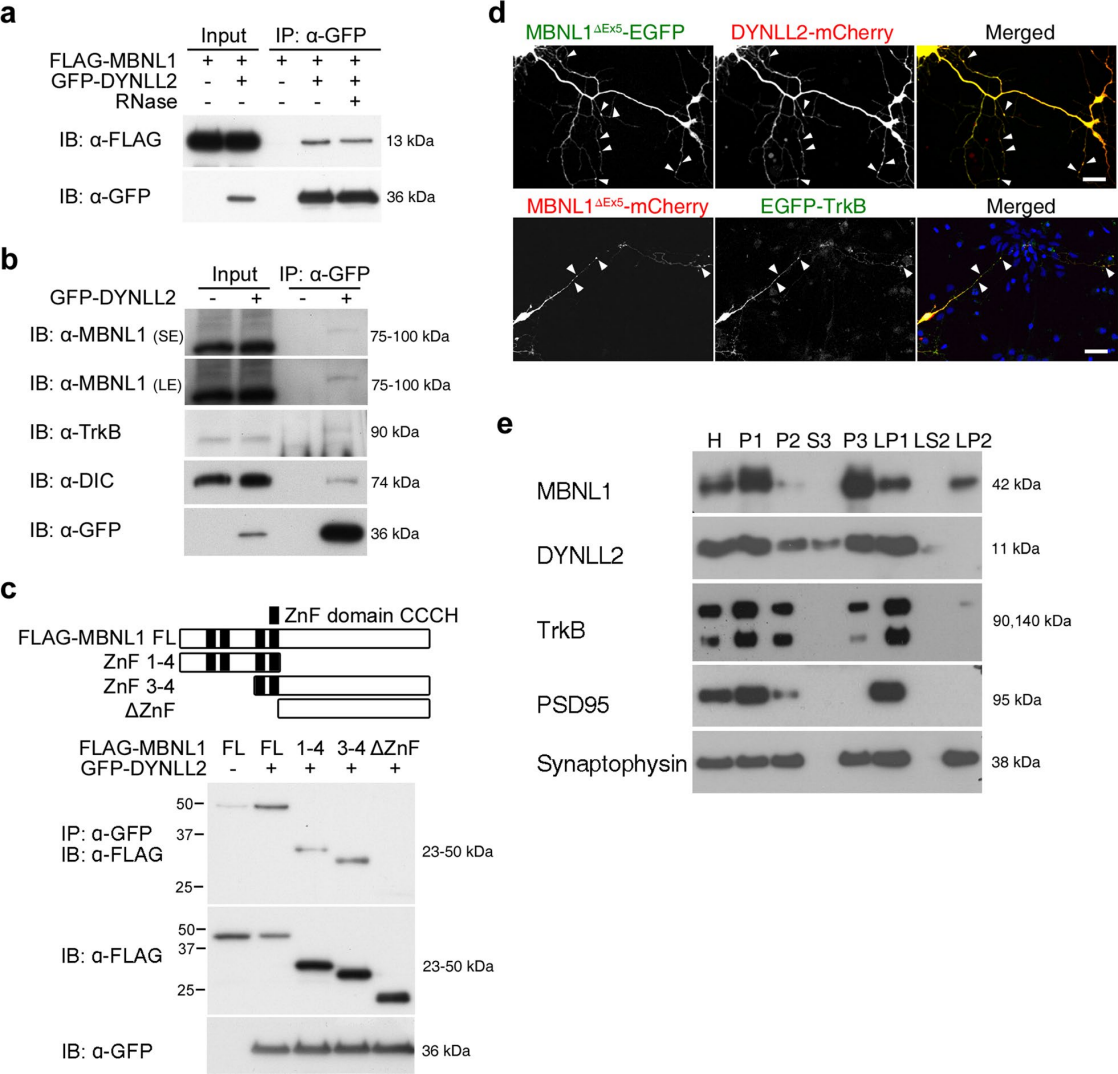
MBNL1 interacts with cytoplasmic DYNLL2 in an RNA-independent manner. (**a**) Examination of FLAG-MBNL1^ΔEx5^ and GFP-DYNLL2 binding by immunoprecipitation using an anti-GFP antibody in Neuro2A cells treated with or without RNase treatment. (**b**) Immunoprecipitation analysis using an anti-GFP antibody to determine binding between endogenous ubiquitinated MBNL1, TrkB and dynein intermediate chain (DIC) in cells expressing GFP-DYNLL2. SE, shorter exposure time. LE, longer exposure time. (**c**) Identification of the DYNLL2 binding domain on MBNL1. Truncated MBNL1 constructs are illustrated on top. An anti-GFP antibody was used to perform immunoprecipitation. ZnF, zinc-finger domains. (**d**) Examination of the distribution of cytoplasmic MBNL1 and DYNLL2 (top) or TrkB (bottom) in neurons using plasmids expressing EGFP-tagged or mCherry-tagged MBNL1^ΔEx5^ with DYNLL2-mCherry or EGFP-TrkB. Arrowheads indicate colocalization region along the neurite. (**e**) Distribution of MBNL1, TrkB and DYNLL2 proteins in the homogenate (H), nuclei and other large debris (P1), crude synaptosomal fraction (P2), cytosolic soluble fraction (S3), light membrane fraction (P3), synaptosomal membrane fraction (LP1), soluble fraction (LS2), and crude synaptic vesicle fraction (LP2). PSD95 and synaptophysin were used as markers for synaptic compartments. Scale bar: **d**, 20 μm

To determine how MBNL1^ΔEx5^ regulates BDNF signaling, we identified MBNL1-interacting proteins by performing an immunoprecipitation (IP) experiment followed by liquid chromatography mass spectrometry (LC–MS/MS) analysis, which revealed cytoplasmic dynein light chain LC8-type 2, DYNLL2 [26], an accessory component of the cytoplasmic dynein complex, among others. Binding of BDNF triggers dimerization and autophosphorylation of TrkB receptor [27]. Retrograde transport of BDNF-TrkB signaling endosomes from the synaptic terminal to the cell body is regulated by the cytoplasmic dynein complex [27, 28]. To confirm the interaction of MBNL1 with DYNLL2, we transiently transfected FLAG-tagged MBNL1^ΔEx5^ (FLAG-MBNL1^ΔEx5^) and GFP-tagged DYNLL2 (GFP-DYNLL2) into Neuro2A cells before performing an IP experiment using anti-GFP antibody. We observed that MBNL1 was immunoprecipitated with the GFP-tagged DYNLL2 (Fig. 2a). This interaction between MBNL1 and DYNLL2 was not affected by RNase treatment, indicating that the interaction is RNA-independent. In addition, upon overexpressing GFP-DYNLL2 in Neuro2A cells for subsequent IP with anti-GFP antibody, we observed that endogenous MBNL1 was again immunoprecipitated (Fig. 2b). And MBNL1 with molecular weight ~ 100 kDa indicates a polyubiquitinated form [7]. Moreover, endogenous TrkB receptor and components of the cytoplasmic dynein transport machinery, including dynein intermediate chain (DIC), were also immunoprecipitated, indicating that MBNL1 and TrkB are associated with the cytoplasmic dynein complex (Fig. 2b). We also investigated the domain in MBNL1 responsible for binding DYNLL2. To do so, we co-transfected constructs expressing GFP-DYNLL2 with different FLAG-tagged MBNL1 fragments with or without zinc-finger domains (FLAG-MBNL1-ZnF1-4, FLAG-MBNL1-ZnF3-4 or FLAG-MBNL1-ΔZnF) and then conducted an IP assay, which revealed that the zinc-finger domains of MBNL1 are required for its interaction with DYNLL2 (Fig. 2c).

Since the cytoplasmic dynein complex is important for retrograde transport of the BDNF-TrkB complex, next we examined if DYNLL2 also plays a role in retrograde transport of TrkB receptor. We found that anti-FLAG antibody could also immunoprecipitate endogenous TrkB receptor from Neuro2A cells overexpressing FLAG-DYNLL2 (Additional File 1a). Overexpressed FLAG-DYNLL2 colocalized with EGFP-tagged TrkB (EGFP-TrkB) in the cell bodies, axonal processes and axon terminals of cultured hippocampal neurons (Additional File 1b). To assess if DYNLL2 is required for BDNF-promoted neurite outgrowth, we examined BDNF responsiveness in DYNLL2-knockdown neurons. We observed that BDNF treatment enhanced axon outgrowth in neurons transfected with a control vector, but DYNLL2-depleted neurons did not respond to BDNF treatment in terms of enhanced axon outgrowth (Additional File 1c), indicating that DYNLL2 is required for regulating BDNF signaling. As GFP-DYNLL2 was associated with MBNL1 and TrkB, we then examined the interaction of MBNL1 and TrkB receptor. We transiently transfected EGFP-TrkB and FLAG-MBNL1^ΔEx5^ into Neuro2A cells. Using anti-GFP antibody for IP, we found FLAG-MBNL1^ΔEx5^ was immunoprecipitated, that indicates an interaction (Additional File 1d ).

Next, we determined the localization and distribution of MBNL1, DYNLL2, and TrkB receptor. We overexpressed EGFP-tagged MBNL1^ΔEx5^ (MBNL1^ΔEx5^-EGFP) and mCherry-tagged DYNLL2 (DYNLL2-mCherry) in cultured hippocampal neurons and observed that both colocalized in axonal processes and terminal regions (Fig. 2d top, arrowhead). Similarly, both overexpressed EGFP-TrkB and mCherry-tagged MBNL1^ΔEx5^ (MBNL1^ΔEx5^-mCherry) colocalized in axonal processes and terminal regions (Fig. 2d bottom). We further examined the subcellular distributions of MBNL1, DYNLL2, and TrkB using biochemical fractionation of adult mouse brain to determine if they occurred in the same fractions. This experiment uncovered a broad subcellular distribution of MBNL1 in the nuclear (P1) and membrane fractions, including P2 (crude synaptosomal fraction), P3 (light membrane fraction), postsynpatic LP1 (synaptosomal membranes), and presynaptic LP2 (crude synaptic vesicle fraction) [23] (Fig. 2e), with PSD95 and synaptophysin being used as markers for the postsynaptic and presynaptic fractions, respectively. DYNLL2 and TrkB were distributed in the P1, P3 and LP1, but not LP2, fractions. Notably, MBNL1, DYNLL2, and TrkB occurred in the LP1 but not LP2 fractions, implying their interaction in the postsynaptic region. These results show that MBNL1 interacts with DYNLL2 in an RNA-independent manner and MBNL1-DYNLL2-TrkB protein complexes are distributed in the postsynaptic region.

### Loss of MBNL1 impairs DYNLL2 motility

**Fig. 3:**
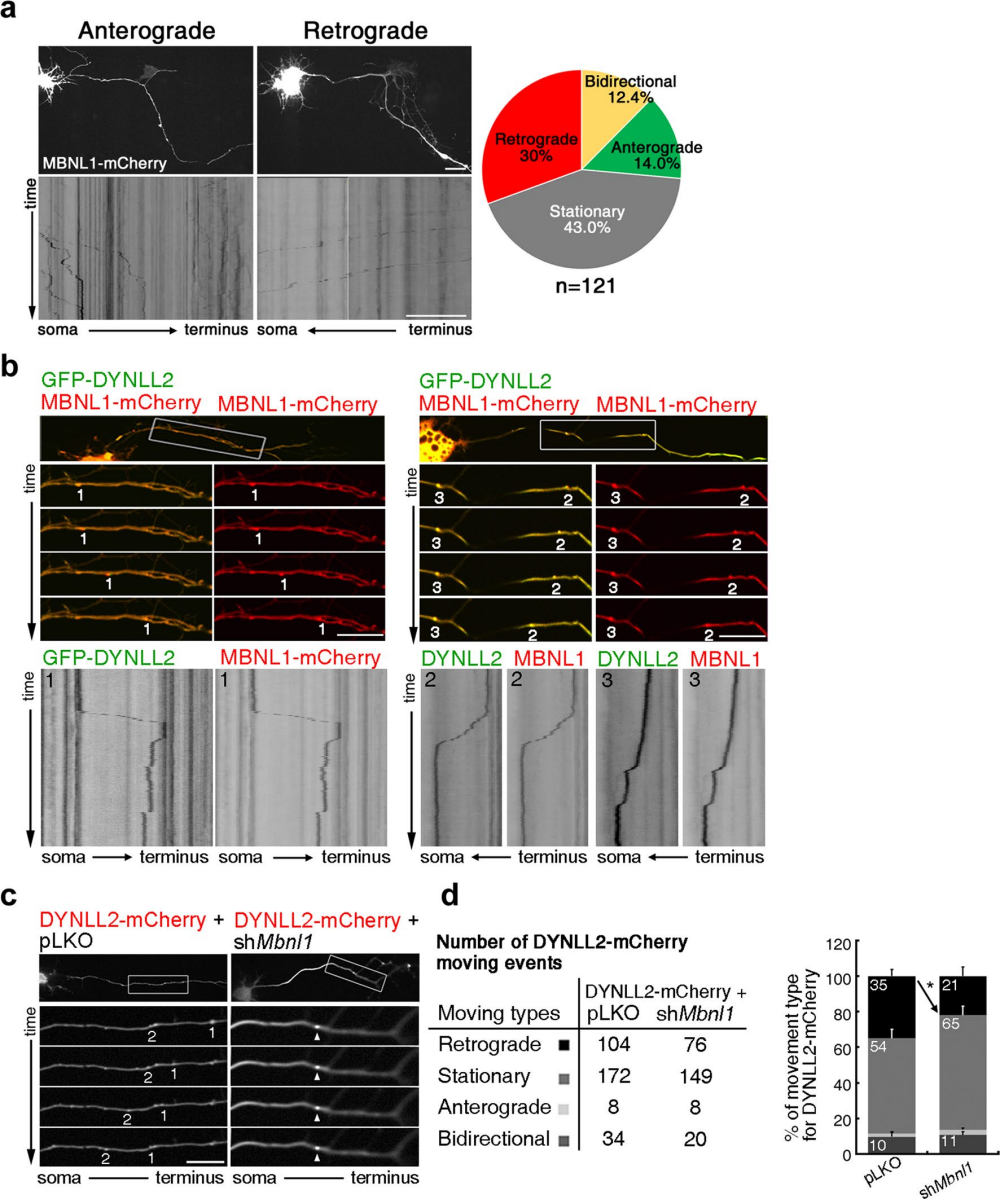
Loss of MBNL1 impairs DYNLL2 motility in neurons. (**a**) Time-lapse imaging of the MBNL1 movement in neurons expressing mCherry-tagged MBNL1. A respective kymograph is shown at the bottom. Quantification of the indicated types of movement from a total of 121 neurons is shown at right. (**b**) Movement of MBNL1^ΔEx5^ and DYNLL2 in neuronal axons. Anterograde (marked “1”), retrograde (marked “2”) and stationary (marked “3”) movements have been labeled. (**c**) Time-lapse analysis showing the movement of overexpressed DYNLL2 in MBNL1-depleted neurons. Neurons were transfected with DYNLL2-mCherry, *Mbnl1* shRNA (sh*Mbnl1*) or control shRNA (pLKO) as indicated. (**d**) Number of DYNLL2-mCherry movement events categorized into 4 different movement types was listed. Quantification of different DYNLL2-mCherry movements in the control and MBNL1-depleted neurons. Stationary DYNLL2-mCherry is indicated by an arrowhead. Scale bars: **a-c**, 40 μm. Data are mean ± SEM. *P < 0.05 by Student’s *t*-test

The broad distribution of MBNL1 indicates that it is highly mobile. We wondered if and how MBNL1 and DYNLL2 may regulate each other’s movements. First, we examined the motility of MBNL1^ΔEx5^ in cultured hippocampal neurons by means of time-lapse imaging. We observed mCherry-tagged MBNL1^ΔEx5^ (MBNL1 ^ΔEx5^-mCherry) moving in: (i) an anterograde direction, i.e., from the soma toward the distal axon; (ii) a retrograde direction, i.e., from the distal axon toward the soma; or (iii) bidirectionally within a certain range (Fig. 3a and Additional File 2). Moreover, a notable percentage of MBNL1 remained stationary along axonal processes. Upon co-expressing mCherry-tagged MBNL1^ΔEx5^ (MBNL1 ^ΔEx5^-mCherry) and GFP-tagged DYNLL2 (GFP-DYNLL2) in cultured hippocampal neurons, we observed that MBNL1-mCherry and GFP-DYNLL2 moved together along axonal processes, either toward the terminal region (Fig. 3b, left, marked with “1”) or the soma (Fig. 3b, right, marked with “2”) or remained stationary (Fig. 3b, marked with “3”).

Then, we knocked down MBNL1 or DYNLL2 and determined how the motility of the other molecule was affected. First, we assayed DYNLL2 motility in MBNL1-depleted neurons. Most DYNLL2 exhibited retrograde movement toward the soma upon BDNF stimulation in neurons co-transfected with control vector (pLKO) and DYNLL2-mCherry, (Fig. 3c, left, Additional File 3). However, in the MBNL1-knockdown neurons, DYNLL2-mCherry movements were significantly retarded and indeed appeared to become immobile (Fig. 3c, right, arrowhead), resulting in a reduced percentage of retrograde movement (Fig. 3d). In contrast, MBNL1 motility in DYNLL2-depleted neurons was not affected, with MBNL1-mCherry moving toward the soma or synaptic terminal region (Additional File 4). Thus, our results show that the cytoplasmic isoform of MBNL1 interacts with DYNLL2 and that MBNL1 depletion impairs the retrograde movement of DYNLL2 in response to BDNF stimulation.

### Loss of BDNF-TrkB signaling in the DM1 mouse brain and the human postmortem DM1 brain

**Fig. 4:**
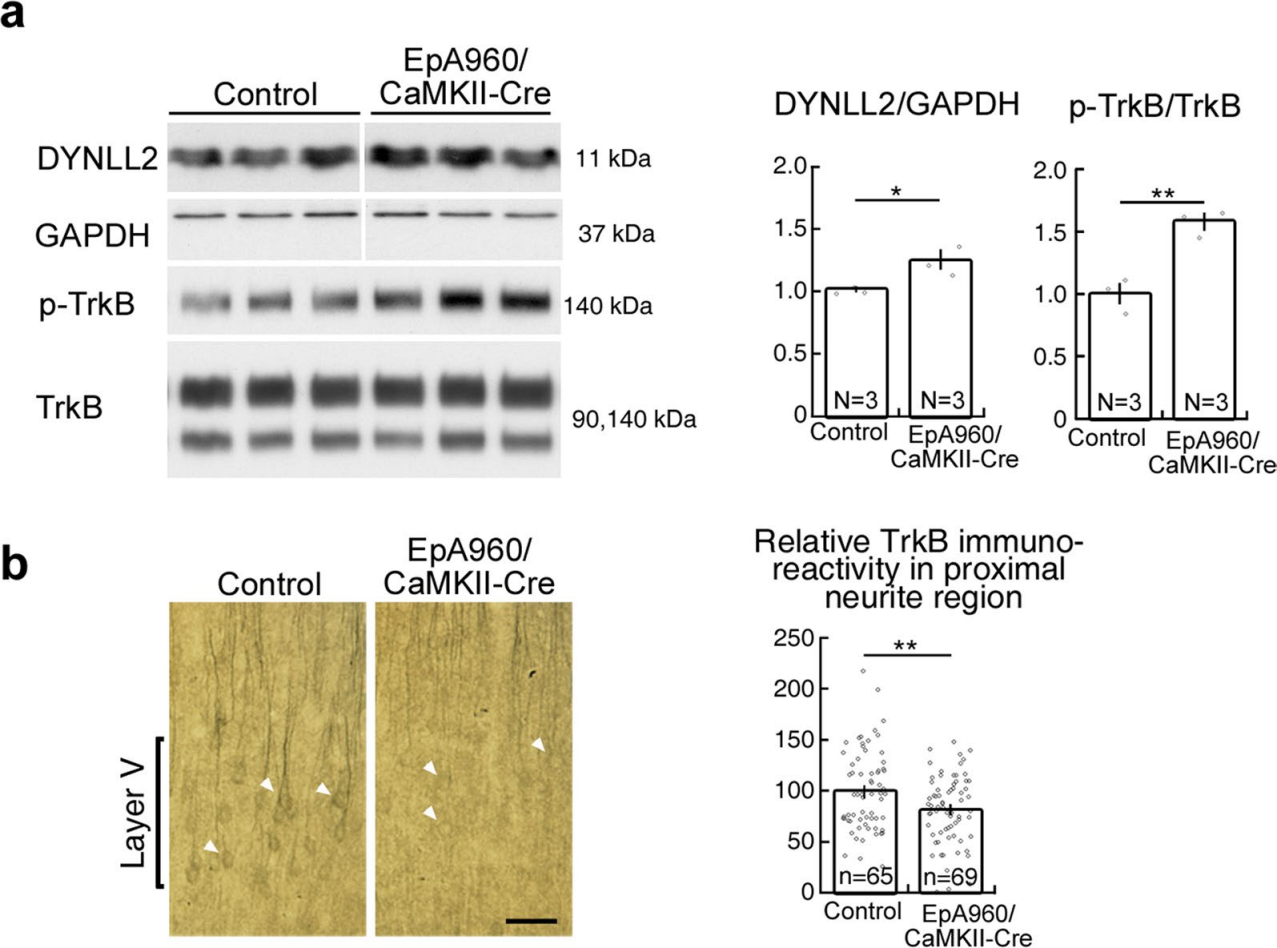
Impaired retrograde transport of BDNF-TrkB signaling in the DM1 mouse brain. (**a**) Expression levels of DYNLL2 and p-TrkB in the EpA960/CaMKII-Cre and control mouse brains. The control group includes two non-Tg and one EpA960 animals. Relative amounts of DYNLL2 and p-TrkB are normalized according to GAPDH and TrkB, respectively. (**b**) Representative TrkB immunoreactivity of cortical-layer V neurons in EpA960/CaMKII-Cre mice at age 6 months. Quantification of the intensity of TrkB immunoreactivity in the proximal neurite region. TrkB immunoreactivity in the cell body is indicated by arrowhead. V, layer V neurons. Number of animals (N) and number of neurons (n) in each group is indicated. Data are mean ± SEM. *p < 0.05, **p < 0.01 by Student’s *t*-test. Scale: 50 μm

Next, we determined if retrograde BDNF-TrkB signaling is impaired in EpA960/CaMKII-Cre brains. We have previously demonstrated that deubiquitination-mediated MBNL1 nuclear translocation results in a reduced cytoplasmic fraction of MBNL1 [7]. Given that the percentage of DYNLL2 retrograde movement was reduced in MBNL1-depleted neurons, we wondered if a reduced cytoplasmic fraction of MBNL1 would result in DYNLL2 accumulation in the synaptic region. Accordingly, we determined the expression of DYNLL2 in the LP1 fraction and indeed observed increased DYNLL2 levels in the LP1 fraction of EpA960/CaMKII-Cre brains (Fig. 4a). Furthermore, the levels of phospho-TrkB, the active form of the TrkB receptor upon BDNF binding, were increased. Examination of the distribution of TrkB immunoreactivity in cortical region of EpA960/CaMKII-Cre brains also revealed a reduced expression within the proximal neurite region, supporting impaired retrograde transport of the DYNLL2-TrkB complex (Fig. 4b).

**Fig. 5:**
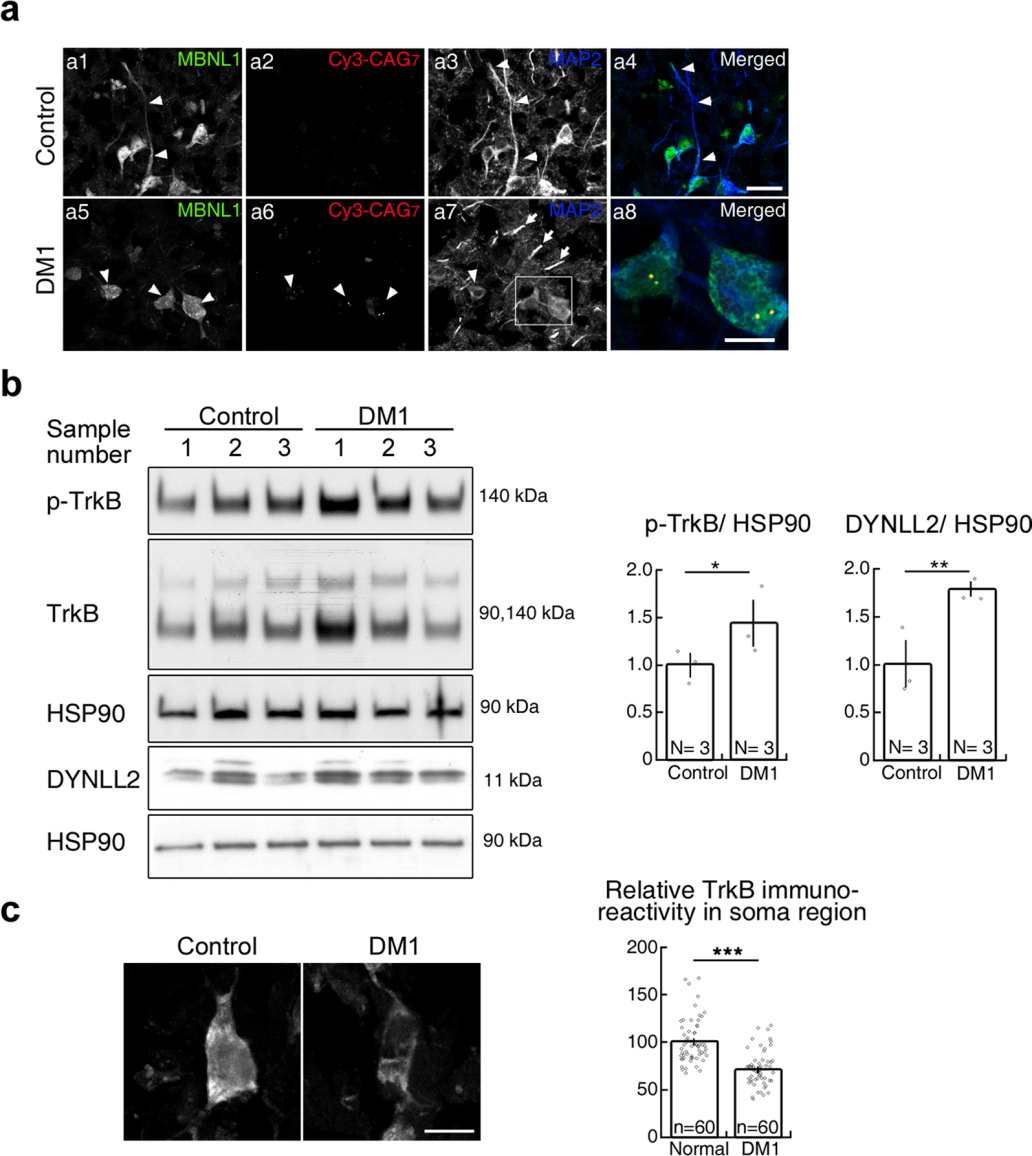
Impaired retrograde transport of BDNF-TrkB signaling in human postmortem DM1 brain. (**a**) Examination of cytoplasmic MBNL1 and dendrite integrity in the cerebral cortex of individual with DM1. Neuron expressing MBNL1 was marked with arrowhead. Fragmented dendrites are indicated by arrows (**a**7). (**a**8) Enlarged image from the inset in a7 shows the colocalization of MBNL1 with RNA foci in the nucleus. (**b**) Examination of p-TrkB and DYNLL2 levels in the P2 fraction from postmortem brain tissues of normal and DM1 individuals. HSP90 was detected as a loading control. (**c**) Representative TrkB immunoreactivity in the cell body of cortical neurons in DM1 brain. Number of human brain (N) and number of neurons (n) in each group is indicated. Data are presented as mean ± SEM. *p < 0.05, **p < 0.01, ***P < 0.001 by Student’s *t*-test. Scale: a1-7, 50 μm; a8, 10 μm; c, 20 μm

Finally, we determined if MBNL1-deficiency-impaired retrograde BDNF-TrkB transport also occurs in human postmortem DM1 brains. To do so, first we examined the distribution of MBNL1 in postmortem DM1 brains. In the cerebral cortex of age-matched normal brain, MBNL1 was localized in dendrites and the cell body, and dendrites with MAP2 staining were relatively intact (Fig. 5a1-4). However, in DM1 brains, MBNL1 was barely detected in dendrites and mainly localized in the nucleus, and a fraction of nuclear MBNL1 was colocalized with RNA foci (Fig. 5a8). In addition, dendrites with MAP2 staining were fragmented (Fig. 5a7, arrow), consistent with our findings in EpA960/CaMKII-Cre brains [11].

Next we examined the expression levels of phospho-TrkB and DYNLL2 in the crude synaptosomal (P2) fraction of the brains of DM1 patients. We found that the expression levels of phospho-TrkB and DYNLL2 were increased in the DM1 patient brains (Fig. 5b). We noticed that the level of total TrkB expression was increased in some samples, and to avoid a possibility of unequal loading, therefore we used HSP90, a marker of P2 fraction, as the loading control. Furthermore, examination of the distribution of TrkB receptor in DM1 patient brains showed a reduction of TrkB immunoreactivity in the soma region (Fig. 5c), that indicates impaired retrograde transport of DYNLL2-TrkB complex. Thus, phospho-TrkB receptor accumulation in the synaptic region indicates that impaired retrograde movement for BDNF-TrkB signaling contributes to DM1. In summary, we show that MBNL1 mediates BDNF-TrkB retrograde signaling by interacting with dynein light chain DYNLL2. Loss of the cytoplasmic function of MBNL1 elicits loss of BDNF responsiveness and morphological impairment due to impaired retrograde transport of BDNF-TrkB via DYNLL2.

## Discussion

Similar to other neurological diseases, the decline in cognitive function typical of DM1 is associated with brain atrophy, neurotransmission and metabolic dysfunctions, and aberrant connectivity [29]. How expanded CUG RNA elicits disease phenotypes remains largely unclear, especially in terms of the structural abnormalities involved in global cortical volume loss [30, 31]. Loss of MBNL-regulated functions has been implicated as contributing to DM1-linked neural pathogenesis, yet how dysfunctional MBNL proteins elicit features of the DM1 brain also remains elusive. In the present study, we investigated how loss of MBNL1 cytoplasmic function induces changes in neuronal morphology. We provide evidence that loss of MBNL1 impairs BDNF-TrkB retrograde signaling, thereby contributing to structural abnormalities in neurons expressing *DMPK*-CUG^exp^. We show that depletion of MBNL1 from cultured hippocampal neurons results in loss of responsiveness to exogeneous BDNF. Using IP experiments, we have revealed that MBNL1 interacts with DYNLL2 and is associated with the cytoplasmic dynein complex. Using time-lapse imaging, we observed co-movement of overexpressed MBNL1 and DYNLL2 along axonal processes, and that the percentage of retrograde DYNLL2 movement was reduced in MBNL1-knockdown neurons. Thus, loss of BDNF responsiveness in MBNL1-depleted neurons is due to impaired retrograde transport of DYNLL2. As RNA-binding proteins, the MBNL proteins act as important regulators of alternative splicing and polyadenylation, as well as mRNA transport. Our findings reveal an unappreciated role for MBNL1 beyond RNA-binding in regulating BDNF-TrkB signaling, with MBNL1 potentially functioning as an adaptor that interacts with DYNLL2 to provide an additional layer of regulation for dynein-mediated TrkB retrograde transport.

Dysregulated expression of BDNF has been reported for a number of neurological disorders [16, 20, 32]. We also observed reduced levels of mature BDNF protein in DM1 mouse brain. Although the causal mechanism underlying reduced BDNF expression remains elusive, it has previously been reported that levels of *Bdnf* mRNA are reduced in the prefrontal cortex of *Mbnl2*-knockout brains [10], indicating that MBNL2 may be involved in regulating *Bdnf* mRNA. In our study, *DMPK*-CUG^exp^ induced impaired retrograde BDNF-TrkB signaling, rendering BDNF supplementation an inappropriate therapeutic strategy for DM1. Recently, a modified version of MBNL1 (MBNL1Δ), in which the N-terminal domain that encompasses RNA-binding domains ZNF1 to 4, was developed to serve as a decoy for *DMPK*-CUG^exp^, thereby releasing endogenous MBNL1 from *DMPK*-CUG^exp^ RNA [33]. Delivery of this modified MBNL1 decoy via an adeno-associated virus (AAV) into the skeletal muscle of a DM1 mouse model significantly ameliorated the disease phenotype, representing a promising approach for DM1 therapy. In our study, we uncovered that MBNL1 interacts with DYNLL2 via the RNA-binding domain of the former. Thus, it warrants investigation if AAV-mediated delivery of MBNL1Δ may be deployed as a DM1 therapy and if MBNL1Δ sequesters endogenous DYNLL2.

It has been suggested that cargo binding specificity of dynein complex is regulated by non-motor subunit dynein light chains, which bind directly or indirectly to the motor heavy chain tails [34]. DYNLL2 plays a role in stabilizing the postsynaptic scaffold protein complex to enhance NMDA signaling [35]. The DYNLL2 homolog in Drosophila, *cut up (Ctp)*, is involved in proteasome trafficking but not in the transport of synaptic vesicle protein [36]. In our study, we have shown that DYNLL2 acts in BDNF-TrkB signaling by interacting with TrkB. We have also shown that DYNLL2 knockdown did not affect MBNL1 motility, that supports its role as a non-motor subunit of the dynein complex and suggests a different regulation for controlling MBNL1 motility. In addition to impaired retrograde BDNF signaling, misregulated alternative splicing of *GRIP1* exon 21 in DM1 brain has been shown to impair binding of GRIP1 to kinesin KIF5A [37], implying perturbed kinesin-mediated intracellular trafficking. Thus, *DMPK*-CUG^exp^ may also affect intracellular trafficking and transport, ultimately impairing neuronal morphology and connectivity.

## Supplementary Information

**Additional file 1.** **Loss of cytoplasmic MBNL1 expression impairs responsiveness to BDNF supplementation in neurons.** (**a**) Examination of BDNF levels by Western blotting analysis in brains from control and EpA960/CaMKII-Cre mice aged one to 1.5 years. Three non-Tg and one EpA960 mice were included as controls. Quantification of BDNF levels, normalized with GAPDH, are shown at right. (**b**, **c**) Effect of BDNF treatment on axon development in neurons expressing Dmpk-CUG^960^ mRNA or upon MBNL1 depletion. (**d**) Effect of cytoplasmic MBNL1 overexpression on neuronal responsiveness to BDNF treatment. Neurons were transfected with plasmids expressing Dmpk-CUG^960^ mRNA (DMPK-CUG^960^) or Dmpk mRNA control (DMPK-CUG^0^) in (**b**), with plasmids expressing Mbnl1 shRNA (pLKO-shMbnl1) or control shRNA (pLKO) in (**c**), or with plasmids expressing Dmpk-CUG^960^ plus cytoplasmic MBNL1 (FLAG-MBNL1^ΔEx5^) or Dmpk mRNA control alone in (**d**). Plasmid expressing GFP was co-transfected to label neuronal morphology. Quantification of total axon length is shown at right. Numbers of neurons (n, from three independent cultured neuronal preparations and transfections) used for quantification are indicated. Data are mean ± SEM. *p<0.05, **p<0.01, ***p<0.001, by Student’s t-test (**a**) or one-way ANOVA with Holm-Sidak test (**b**-**d**). Scale bar: **b**-**d**, 100 µm.

**Additional file 2. Representative video of the time-lapse imaging of overexpressed MBNL1**^**ΔEx5**^**-mCherry in neurons.**

**Additional file 3.** **Representative video of the time-lapse imaging of DYNLL2-mCherry in the control and MBNL1-depleted neurons.**

**Additional file 4.** **Loss of DYNLL2 do not affect the MBNL1 motility in neurons.**Time-lapse analysis showing the movement of overexpressed MBNL1 in DYNLL2-depleted neurons. Arrow indicates the retrograde movement of MBNL1-mCherry. Arrowhead represents the anterograde movement of MBNL1-mCherry. Scale: 40 µm.
