# Supplementary figures and images for: Loss of MBNL1-mediated retrograde BDNF signaling in the myotonic dystrophy brain
Source: Acta Neuropathol Commun. 2023 Mar 15;11:44. doi: 10.1186/s40478-023-01540-x (PMC10018927; doi:10.1186/s40478-023-01540-x)

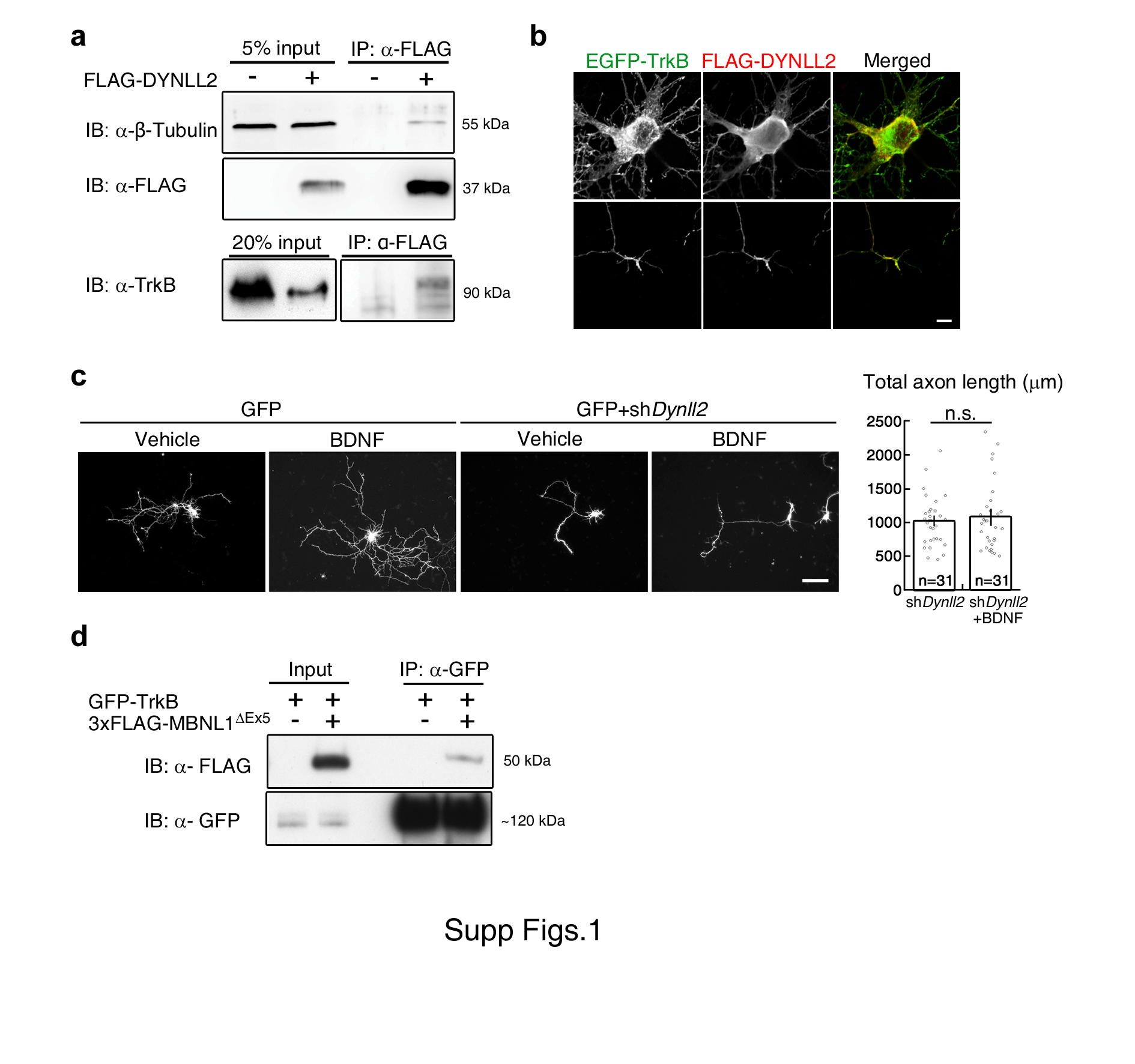

Supplement: Supplementary file 1 — Additional file 1. Loss of cytoplasmic MBNL1 expression impairs responsiveness to BDNF supplementation in neurons. (a) Examination of BDNF levels by Western blotting analysis in brains from control and EpA960/CaMKII-Cre mice aged one to 1.5 years. Three non-Tg and one EpA960 mice were included as controls. Quantification of BDNF levels, normalized with GAPDH, are shown at right. (b, c) Effect of BDNF treatment on axon development in neurons expressing Dmpk-CUG960 mRNA or upon MBNL1 depletion. (d) Effect of cytoplasmic MBNL1 overexpression on neuronal responsiveness to BDNF treatment. Neurons were transfected with plasmids expressing Dmpk-CUG960 mRNA (DMPK-CUG960) or Dmpk mRNA control (DMPK-CUG0) in (b), with plasmids expressing Mbnl1 shRNA (pLKO-shMbnl1) or control shRNA (pLKO) in (c), or with plasmids expressing Dmpk-CUG960 plus cytoplasmic MBNL1 (FLAG-MBNL1ΔEx5) or Dmpk mRNA control alone in (d). Plasmid expressing GFP was co-transfected to label neuronal morphology. Quantification of total axon length is shown at right. Numbers of neurons (n, from three independent cultured neuronal preparations and transfections) used for quantification are indicated. Data are mean ± SEM. *p<0.05, **p<0.01, ***p<0.001, by Student’s t-test (a) or one-way ANOVA with Holm-Sidak test (b-d). Scale bar: b-d, 100 µm. [file 40478_2023_1540_MOESM1_ESM.tif]

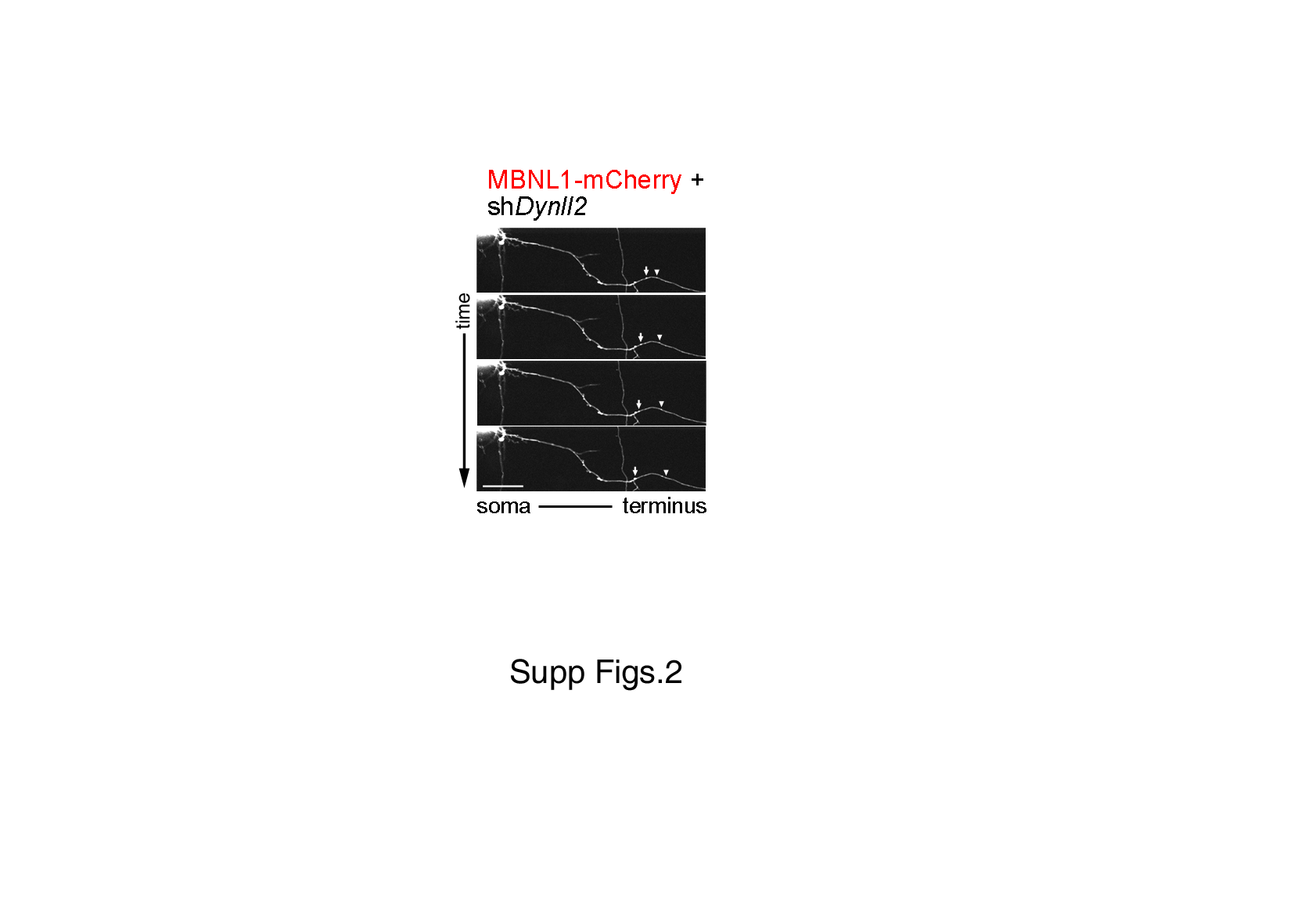

Supplement: Supplementary file 4 — Additional file 4. Loss of DYNLL2 do not affect the MBNL1 motility in neurons.Time-lapse analysis showing the movement of overexpressed MBNL1 in DYNLL2-depleted neurons. Arrow indicates the retrograde movement of MBNL1-mCherry. Arrowhead represents the anterograde movement of MBNL1-mCherry. Scale: 40 µm. [file 40478_2023_1540_MOESM4_ESM.tif]
